# MapB Protein is the Essential Methionine Aminopeptidase in *Mycobacterium tuberculosis*

**DOI:** 10.3390/cells8050393

**Published:** 2019-04-28

**Authors:** Miriam Vanunu, Patrick Schall, Tali-Haviv Reingewertz, Pradip K. Chakraborti, Bernhard Grimm, Daniel Barkan

**Affiliations:** 1 Koret School of Veterinary Medicine, Robert H. Smith Faculty of Agriculture, Nutrition and Environment, The Hebrew University of Jerusalem, Rehovot 76100, Israel; mirivanunu@gmail.com (M.V.); tali.reingewertz@mail.huji.ac.il (T.-H.R.); 2 Humboldt-Universität zu Berlin, Institute of Biology/Plant Physiology, Philippstr.13, Building 12, 10115 Berlin, Germany; schallpa@hu-berlin.de (P.S.); bernhard.grimm@rz.hu-berlin.de (B.G.); 3 Department of Biotechnology, School of Chemical and Life Sciences, Jamia Hamdard, Hamdar Nagar, New Delhi 110062, India; pradipkchakraborti@gmail.com

**Keywords:** Methionine aminopeptidase, tuberculosis, mycobacteria, gene essentiality, ribosome

## Abstract

*M. tuberculosis (Mtb)*, which causes tuberculosis disease, continues to be a major global health threat. Correct identification of valid drug targets is important for the development of novel therapeutics that would shorten the current 6–9 month treatment regimen and target resistant bacteria. Methionine aminopeptidases (MetAP), which remove the N-terminal methionine from newly synthesized proteins, are essential in all life forms (eukaryotes and prokaryotes). The MetAPs contribute to the cotranslational control of proteins as they determine their half life (N-terminal end rule) and facilitate further modifications such as acetylation and others. Mtb (and *M. bovis*) possess two MetAP isoforms, MetAP1a and MetAP1c, encoded by the *mapA* and *mapB* genes, respectively. Conflicting evidence was reported in the literature on which of the two variants is essential. To resolve this question, we performed a targeted genetic deletion of each of these two genes. We show that a deletion mutant of *mapA* is viable with only a weak growth defect. In contrast, we provide two lines of genetic evidence that *mapB* is indispensable. Furthermore, construction of double-deletion mutants as well as the introduction of point mutations into *mapB* resulting in proteins with partial activity showed partial, but not full, redundancy between *mapB* and *mapA*. We propose that it is MetAP1c (*mapB*) that is essentially required for mycobacteria and discuss potential reasons for its vitality.

## 1. Introduction

*Mycobacterium tuberculosis*-complex bacteria are a major cause of morbidity and mortality in the developing as well as the developed world [1]. Control of the disease is hindered by the emergence of highly antibiotic-resistant strains as well as by the pathogens’ ability to enter a persistent state where it tolerates conventional antibiotics even in the absence of genetic drug-resistance [2], necessitating long, complicated treatment regimens. Novel drugs are in dire need and the discovery and validation of new drug-targets are therefore of high importance.

Methionine aminopeptidase (MetAP, previously MAP) is a metallo-protease that removes the N-terminal methionine from newly synthesized proteins. This co-translational control mechanism is conserved in all life forms from bacteria to high vertebrates and plants [3]. There are two classes of this enzyme—MetAP1 and MetAP2 and humans possess them both. MetAP1, which is more prevalent in prokaryotes, is further subdivided into four subtypes—MetAP1a, b, c and d [4]. The gram negative bacteria *E. coli* and *S. typhimurium*, for example, have one copy of a MetAP1a and its function was shown to be indispensable for viability [5,6]. *M. tuberculosis* (like other actinobacteria) has two MetAP1 genes: MetAP1a (*mapA*, *Rv0734* in H37Rv, *Mb0755* in *M. bovis*) and MetAP1c (*mapB, Rv2861c, Mb2886c*). The main difference between them is an additional N-terminal stretch of 40 amino acids in MapB which is thought to play a role in the interaction with the ribosome [7]. The essentiality of the MetAP pathway was never formally proven in Mtb, although the outcome of studies with other organisms makes this a likely assumption. Which of the two genes in Mtb is essential or whether they are synthetically lethal are questions derived from conflicting reports: one study [8], using chemical inhibition of both enzymes, showed marked reduction of bacterial viability, however no complete lethality, possibly due to incomplete inhibition. A knock-down of each of these genes separately by expressing antisense RNA suggested that *mapA* (MetAP1a) was more important for viability as a knockdown of *mapB* (MetAP1c) using the same method had only a little effect, suggesting that *mapB* was less important. In contrast, analysis of a high density transposon-mutant library in Mtb [9] suggested that *mapA* was not essential, whereas *mapB* was. A targeted gene deletion experiment to clarify this conundrum was not performed. Since essential genes are an attractive target for drug development (especially genes coding for enzymes that could potentially be inhibited), it is imperative to pinpoint which of these two genes is the essential one in order to focus inhibitor development on the correct isoform [10]. We therefore intended to delete each of the two genes in order to firmly establish which is the essential, whether only the inhibition of both genes/inactivation of both isoforms leads to a lethal phenotype (synthetic lethality), or whether the MetAP pathway is completely non-essential in mycobacteria. We created a double *mapA/mapB* deletion mutant and continued to create point mutations of the genes affecting their activity to test the minimal requirements for viability.

## 2. Materials and Methods

### 2.1. Bacteria and Growth Conditions

We used *M. tuberculosis-complex* bacteria, *M. bovis BCG pasteur*. Bacteria were grown in standard 7H9 media or on 7H10 plates supplemented by 0.05% glycerol, 10% ADS (Albumin-Dextrose-NaCl) and tween80 (for 7H9 only). Antibiotic concentrations for mycobacteria were 50 µg/ml (hygromycin), 20 µg/ml (kanamycin and streptomycin) and 17–25 µg/ml (zeocin). ATc (Anhydrotetracycline) was added when appropriate in concentrations of 50 ng/ml. 

### 2.2. Gene Introduction and Deletion

Plasmid introduction into mycobacteria was performed by electroporation, as previously widely described. Gene deletions were performed by the temperature-sensitive transducing mycobacteriophage technique, also previously described [11]. Briefly, the 600 bp regions flanking the gene of interest were PCR amplified, the sequence was confirmed, cloned on either side of the resistance marker on either pMSG360 (hygromycin) or pMSG360Z (Zeocin) and the resulting plasmid was digested by DraI and AflII, producing a linear piece of the knock-out construct flanked by areas homologous to the temperature-sensitive, TM4-based, phAE87. This linear fragment was electroporated into 42 °C-induced EL350 bacteria (*E. coli* expressing recombineering enzymes, induced at 42 °C) that also carry the phagemid of phAE87. Successful recombinant EL350 were isolated on antibiotic selective plates, the phagemid was extracted by phenol-chloroform, electroporated into *M. smegmatis* bacteria and plated for plaques in permissive 30 °C temperature. Plaques were picked, presence of KO construct was confirmed by PCR and it was amplified to infect target BCG bacteria at 39 °C. After infection, BCG bacteria were plated on antibiotic selective plates (with ATc when needed) and grown at 37 °C in 5% CO_2_ for 3–4 weeks.

The V18G and W255L mutations in *mapB* were introduced by a two-step PCR reaction where the primers in the first reaction contained the desired single nucleotide change. Correct sequence was confirmed by Sanger sequencing. 

### 2.3. Expression and Purification of Recombinant MapB Proteins

Wild-type *mapB* and the mutant *mapB^V18G, W255L^* were cloned with a HIS-tag at their N-termini into the pET29a expression vector. The wild type protein was expressed and purified as described in "The QIAexpressionist™—A handbook for high-level expression and purification of 6xHis-tagged proteins". Hilden, Germany: Qiagen, June 2013. The recombinant MapB^V18G;W255L^ was expressed and purified as follows: *E. coli* Rosetta cells, harboring the expression plasmid, were grown at 37 °C to an OD_600_ (Optical Density) of 0.5 and then induced by 1 mM IPTG. After Induction, cells were grown at 30 °C for 3 h before collecting the cell pellet by centrifugation at 4 °C for 15 min at 5000 g. Expression of the protein was checked by SDS-PAGE. Purification of the insoluble MapB^V18G;W255L^ mutant from *E. coli* cells: Proteins were purified under denaturing conditions according to the QIAexpressionist protocol (Qiagen) using an 8 M urea buffer and a nickel-nitrilotriacetic acid agarose. After purification, the proteins were applied dropwise to a refolding buffer (400 mM Arg-HCL, 1 mM EDTA, 3 mM reduced glutathione, 0.3 mM oxidized glutathione and 0.1 M Tris, pH 8) for a series of three days at 4 °C with stirring. After three days, the refolding buffer protein mixture was dialyzed against 5 L TBS (150 mM NaCl, 50 mM Tris-HCl, pH 7.6) with SnakeSkin Dialysis Tubing (Thermo fisher) for 12 h at 4 °C. After dialysis, the protein solution was concentrated with Amicon Ultra-4 Centrifugal Filter Units (Merck-Millipore) and examined by SDS-PAGE.

### 2.4. Activity of the Recombinant Proteins Assessment

Activity was assayed with the fluorogenic substrate Met-MCA (Peptide Institute, Inc). The removal of the methionine was continuously measured by fluorescence (excitation wavelengths = 355 nm, emission wavelength 460 nm). Recombinant protein (0.2 µM) was incubated on ice for 10 min in the assays buffer (50 mM Tris-HCL, 1 mM DTT, 1.5 mM MgCl_2_, pH 7.4). The assay was started by adding of the Met-MCA (final concentration 400 µM) substrate to the assay buffer protein mixture and was performed at room temperature. The measurement was done with an F7000 (Hitachi) Flourescence Spectrophotometer.

## 3. Results

### 3.1. mapA (Rv0734, Mb0755) is Dispensible for Bacterial Viability

**Figure 1:**
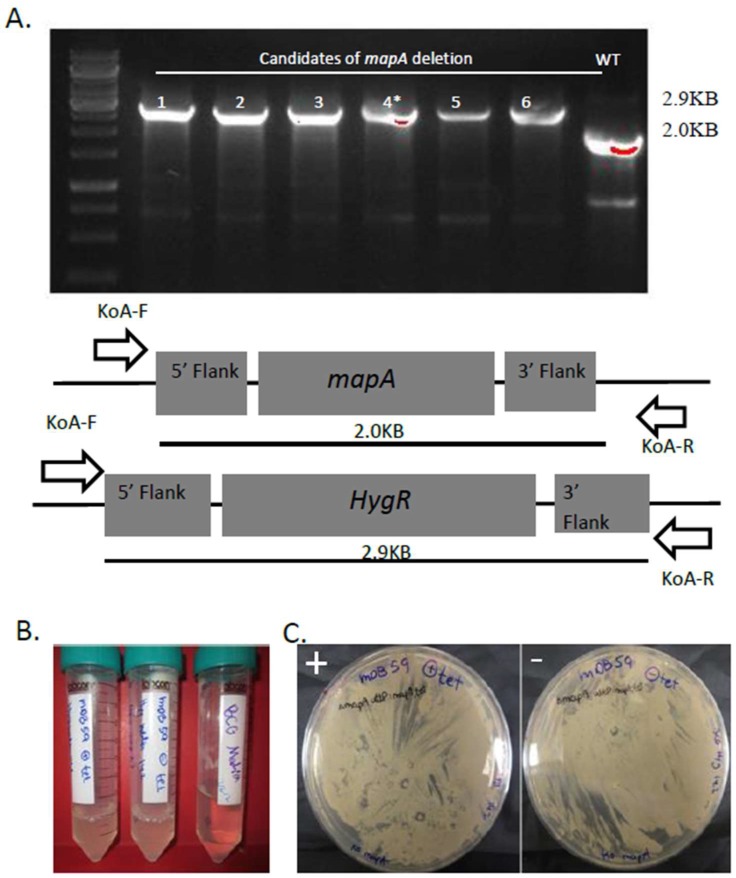
(**A**) Creation and confirmation of a *mapA* deletion mutant. After infection with phDB20, six hygromycin resistant colonies were analyzed by PCR. All six produced a pattern consistent with *mapA* deletion and colony four was named mDB59. (**B**,**C**) mDB59 is not dependent on ATc for growth. (**B**) mDB59 was grown with (leftmost tube) and without (middle tube) ATc (inducing the expression of *mapA*) in liquid. The rightmost tube is clear 7H9 media. (**C**) Left–with ATc, Right–no ATc. No difference in growth is apparent.

In order to examine the essentiality of *mapA*, we took an unbiased approach in which we first pre-complemented wild-type (wt) bacteria with an additional copy of *mapA* at the L5 phage *attB* site (*attB*), creating a mutant with two copies of the gene. To have a better ability to perform depletion studies, the complementing gene was placed under a tetracycline controlled promoter which can be induced by the addition of anhydrotetracycline (ATc), whereas withdrawal of ATc allows only for weak, if any, expression [12]. This construct was placed under kanamycin selection in the plasmid pDB116 and electroporated into BCG-*Pasteur*, creating the mutant mDB25 [*pasteur attb:kan:mapA-ATc*]. We then proceeded to deleting the native *mapA* gene, replacing it with a *hygromycin^R^* cassette, using a specialized transducing phage based on phAE87 (TM4) designated phDB20 [11,13]. After infection with phDB20, bacteria were plated on 7H10 plates with hygromycin and ATc. Six hygromycin resistant colonies appeared and all were picked and analyzed by PCR. A PCR using primers koA-F and koA-R (binding outside of the flanking regions used to create the deletion) would result in a 2.0 kb fragment in wt, however a 2.9 kb fragment in a mutant where successful replacement of *mapA* by *hyg^R^* took place. All six colonies showed a 2.9 kb fragment (Figure 1a); one of them (clone 4) was arbitrarily chosen for further analysis and examined by sequencing. The flanking regions of the native *mapA* on both sides were found, however the *mapA* gene itself, except the first and last 30 bp, was replaced by the *hygromycin^R^* gene. The *mapA* deletion and replacement by *hygromycin^R^* was additionally shown by several digestions of the PCR product of the locus (Supplementary Figure S1). The mutant was called mDB59 [*Pasteur ΔmapA:hygR, attb:kan:mapA-ATc*]. 

**Figure 2:**
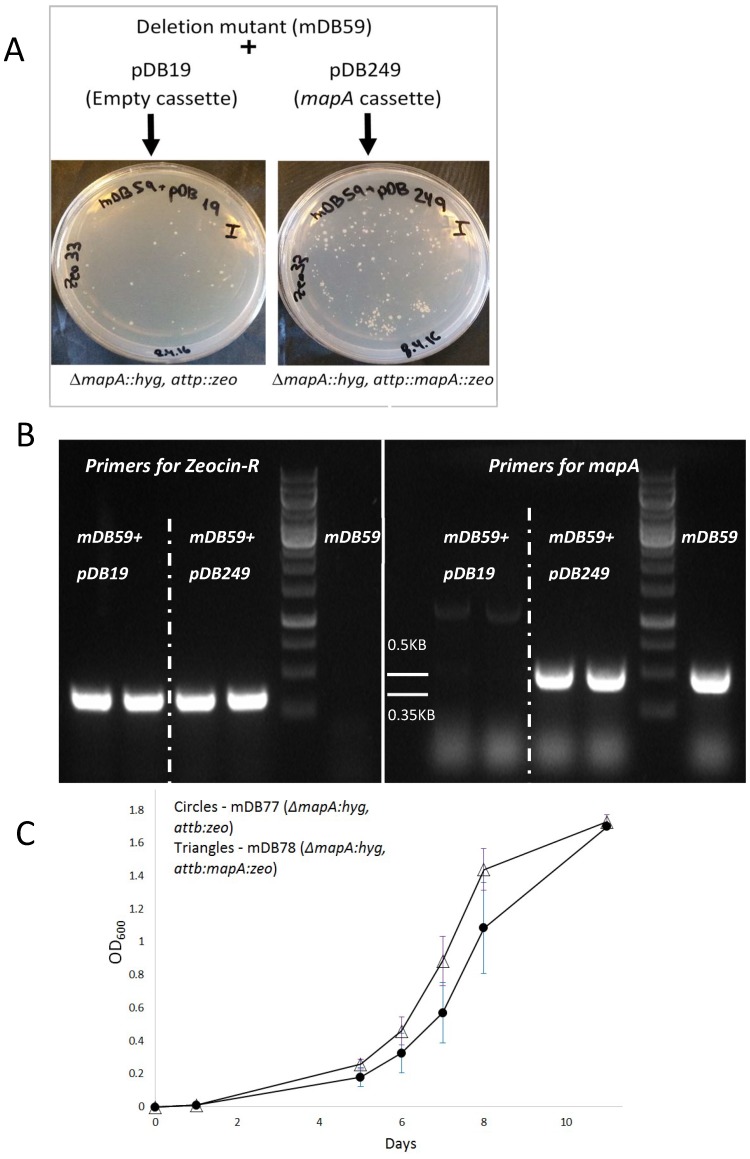
Complete *mapA* deletion mutant. (**A**) Electroporation of mDB59 with either pDB19 or pDB249 (exchanging *mapA-Kan* for *null-zeocin* or *mapA-zeocin*, respectively) both yielded multiple colonies. (**B**) Two random colonies from each electroporation were analyzed by PCR. All four colonies became *zeo-R* positive (left panel, *zeo-R* product is 0.35KB), confirming cassette exchange, however only mDB59 + pDB249 remains positive for *mapA* (Right, expected PCR product of 0.5KB). Both mDB59 + pDB19 colonies are complete *mapA* deletion mutants. (**C**) A complete *mapA* deletion mutant (mDB77) has a mild growth defect in 7H9 media during the exponential phase of growth as compared to mDB78, however it reaches the same final OD (done in duplicates, one of two similar experiments is shown).

To test whether *mapA* was essential, we first opted for transcription silencing by withdrawal of ATc, resulting in abrogation of *mapA* expression. We grew mDB59 in 7H9 media with or without ATc and also plated it on 7H10 plates with and without ATc. Interestingly, and in contrast to the previous reports on *mapA* essentiality, bacteria grew well with or without the addition of ATc—both on 7H10 plates and in 7H9 media (always after repeated washes to remove traces of ATc in media) (Figure 1b,c). This result could be explained by (i) non-essentiality of *mapA*, (ii) (in case *mapA* is essential) by an "escape" of *mapA* from the ATc-controlled expression, or (iii) low-level expression resulting from a leaky promotor control, allowing enough MapA expression for the survival of the bacteria. To unravel the correct possibility, it was intended to completely remove the complementing *mapA* sequence from the bacterial chromosome [14]. For this purpose, we used two additional plasmids: pDB19 (an empty *attP*-integrating plasmid, conferring resistance to zeocin) and pDB249 (the same plasmid containing the *mapA* gene). We separately electroporated both plasmids into mDB59 and plated them on zeocin plates. If *mapA* was essential, we expected to get colonies with pDB249 electroporation only as the exchange for pDB19 would create a null-mutant. Alternatively, if it was not essential, then the exchange of pDB116 for both pDB249 and pDB19 would be possible and we would get zeocin resistant colonies in both electroporations. Indeed, both electroporation yielded dozens of colonies (Figure 2a). We arbitrarily picked two colonies from each plate and examined them by PCR. The results show that all four colonies (two mDB59 + pDB19, two mDB59 + pDB249) acquired the zeocin resistance gene, with the pDB19 or pDB249 replacing the previously integrated cessette (which contained *mapA*). This replacement led to the loss of *mapA* for the two [mDB59 + pDB19] colonies and created a complete *mapA* null strain (Figure 2b). One of the full-deletion colonies was named mDB77 (*ΔmapA;hyg, attb:zeo*), whereas one of the [mDB59 + pDB249] colonies was called mDB78 (*ΔmapA;hyg, attb:mapA:zeo*). The growth of mDB77 (full deletion mutant) was slightly slower during the exponential phase relative to that of mDB78 (Figure 2c). However, the same final OD was reached.

### 3.2. mapB (Rv 2861c, Mb2886c) is Genetically Essential for Bacterial Viability

**Figure 3:**
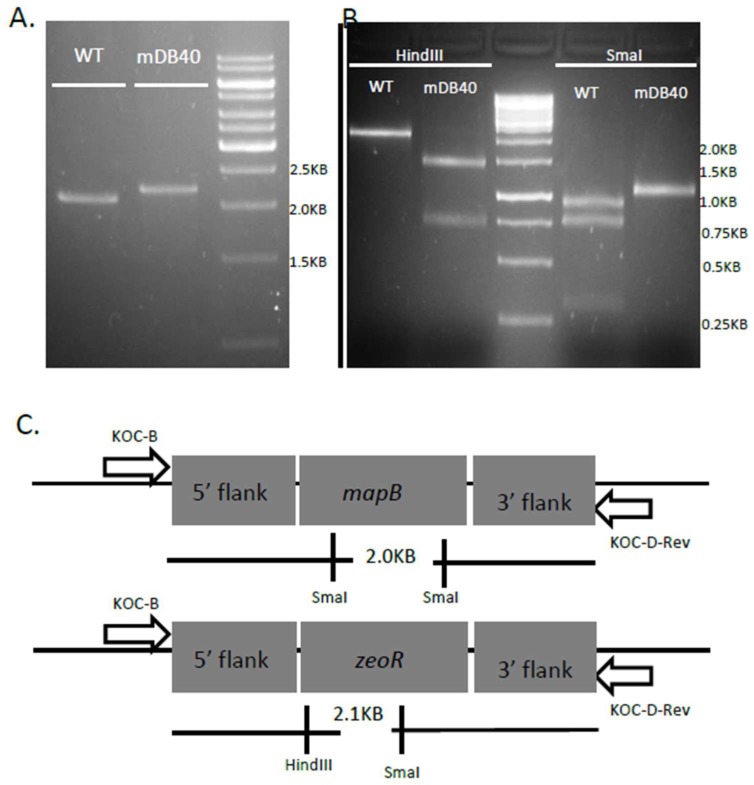
Deletion of the native *mapB*. (**A**) A zeocin resistant colony (mDB40) was analyzed by PCR. In wt, the product size is 2KB, whereas in a correct deletion mutant it is 2.1KB. (**B**) The digestion pattern produced by wt or mDB40 when digested by HindIII (no effect on wt, 1.3, 0.7KB in deletion) or SmaI (0.9, 0.7, 0.3KB in wt, 1.05, 1.05 in deletion mutant) confirms the correct allelic exchange in mDB40 (see diagram in (**C**)).

**Figure 4:**
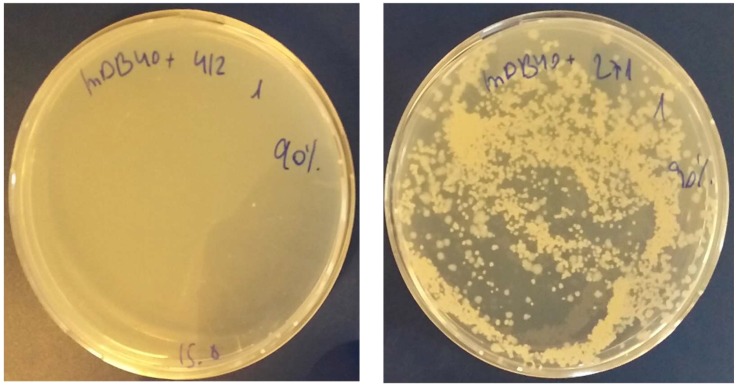
*mapB* is essential in BCG. mDB40 [*pasteur* *ΔmapB:zeo, attb:kana:mapB*] was electroporated by either pYUB412 (*attb:hyg*, left) or pDB271 (*attb:hyg:mapB*, right). Multiple colonies arise with pDB271 where *mapB* is replaced by another copy of the same gene. However, pYUB412 would have yielded a *null-mapB* mutant. No colonies arise on that plate.

As *mapA* was shown not to be essential, we opted for a similar unbiased approach towards *mapB* essentiality. We first pre-complemented BCG *pasteur* with pDB144, which is an attB integrating, kanamycin-selected plasmid, carrying a copy of *mapB* with its native promotor, thus creating mDB26 (BCG *Pasteur attb:mapB:kan*). We then proceeded to delete the native *mapB* gene using the phage phDB13, replacing *mapB* with a *zeocin^R^* cassette. After infection with the phage, bacteria were plated on 7H10 plates with zeocin, and a resistant colony was picked and analyzed in a similar manner to the *mapA* deletion. Because the *zeocin^R^* gene is shorter than the *hygromycin^R^* gene, the PCR product of primers flanking *mapB* (including 700 bases from both sides/flanks) in wt bacteria is only 100bp shorter than the product in a correct deletion mutant (Figure 3a), making a definitive proof of the deletion more difficult, and thus we sent the PCR product for sequencing. The flanking regions of *mapB* were present at both sides of the PCR product, however the *mapB* gene itself was replaced by the *zeocin^R^* gene. Also, replacement of *mapB* by *zeocin^R^* can be distinguished from wt by HindIII and SmaI digestions of the PCR product (Figure 3b). The correct deletion mutant was named mDB40 (*ΔmapB:zeo, attb:mapB:kan*). To examine if *mapB* was essential or not, we again attempted to remove the complementing *mapB* gene from the *attB* site by exchanging it with either one of two plasmids: an empty, hygromycin-selected pYUB412, or the same vector with wt *mapB* on it (pDB271). In contrast to the *mapA* experiment, this time we got multiple colonies only in the electroporation with mDB271 (when *mapB* was simply replaced by another copy), whereas electroporation with pYUB412 yielded no colonies, despite several attempts and long incubation times (Figure 4). One of the clones where *mapB:kana* was replaced by pDB271 (*mapB:hyg*) was called mDB105 (*ΔmapB:zeocin; attB:mapB:hygromycin*) and was used for further experiments. We also conducted a similar experiment, this time with the empty streptomycin-selected vector pDB60 and the same vector with wt *mapB* (pDB231). Again, whereas multiple streptomycin resistant colonies were obtained with pDB231, we did not succeed with pDB60 (Supplementary Figure S2). One of the pDB231-electroporated colonies was called mDB55 (*ΔmapB:zeocin; attB:mapB:streptomycin*), and was also used for further experiments.

To confirm that *mapB* expression was indeed essential, an additional approach was undertaken by using the newly created mDB55 (*ΔmapB:zeo, attb:mapB:strep)*: we constructed a single-copy, kanamycin-selected, episomal plasmid (pDB261), expressing *mapB*. The origin of replication of this plasmid is the MF1 origin, which would be rapidly lost when its presence is not selected by an existing/included antibiotic or another essential gene [15]. The successful introduction of pDB261 into mDB55 was confirmed by PCR and the mutant was called mDB103 (*ΔmapB:zeo, attb:mapB:strep, MF1:mapB:kana*). Then, the *mapB* cassette at the *attb* site was exchanged with either an empty *hygromycin^R^* cassette (pYUB412), creating mDB108 (*ΔmapB:zeo, attb:hyg, MF1:mapB:kana*), or a similar cassette designated pDB271 containing another *mapB* copy, creating mDB107 (*ΔmapB:zeo, attb:mapB:hyg, MF1:mapB:kana*). This approach allowed us to test whether the bacterium can lose its copy of *mapB* if it does not have an additional copy. We grew both mDB107 and mDB108 for 12 doubling times without kanamycin with the option to lose the pDB261 plasmid in case *mapB* is not essential. We then plated for and examined single colonies for kanamycin resistance. As expected, ~75% of the mDB107 colonies [*attb:hyg:mapB*] lost their kanamycin resistance (23 colonies grew on kana + hyg, as opposed to 98 colonies on hygromycin alone) as there was no selection pressure for the retention of pDB261. In contrast, all of mDB108 [*attb:hyg*] mutants retained pDB261 with its kanamycin resistance (86 colonies on kana + hyg and 90 colonies on hygromycin alone), as the pDB261 plasmid was their sole source of *mapB* expression and MapB activity.

These results indicate that in contrast to the previous chemical inhibition report and in agreement with the transposon-mutant library analysis, *mapB*, and not *mapA*, is the essential *MetAP* gene in *M. bovis* BCG (and probably in all *M. tubercu* losis-Complex bacteria).

### 3.3. Creation of a Double Map Mutant

To further explore the role of the two *map* genes and to clarify whether they can compensate for each other, we decided to create a double mutant *ΔmapA ΔmapB*. Because the complete *Δ*mapB mutant is not viable, we started off with the mDB78 mutant, described earlier in this report (*ΔmapA:loxP-hygromycin^R^-loxP; attb:zeocin^R^:mapA)*. We first "cured" mDB78 of the *hygromycin^R^* gene by introducing a kanamycin-selected, MF1-origin plasmid named pMSG381 which has the *cre-recombinase* on it [16]. After the loss of pMSG381 (MF1-origin plasmids are rapidly lost without the antibiotic selection pressure), the *hygromycin^R^*-cured mutant was named mDB122 (*ΔmapA:loxP; attb:zeocin^R^:mapA*). We then changed the *attb* cassette for an additional copy of *mapB* (pDB271), creating the mDB128 strain (*ΔmapA:loxP; attb:hygromycin^R^:mapB)*, and used phDB13 to delete *mapB* from this mutant, creating mDB130 (*ΔmapA:loxP; ΔmapB:zeocin^R^; attb:hygromycin^R^:mapB*). The deletion of the native *mapB* was confirmed using the same methods as the single-deletion *mapB* mutant (mDB40). 

### 3.4. Complementation of ΔmapB Mutants with mapB with Reduced Enzymatic Activity

**Figure 5:**
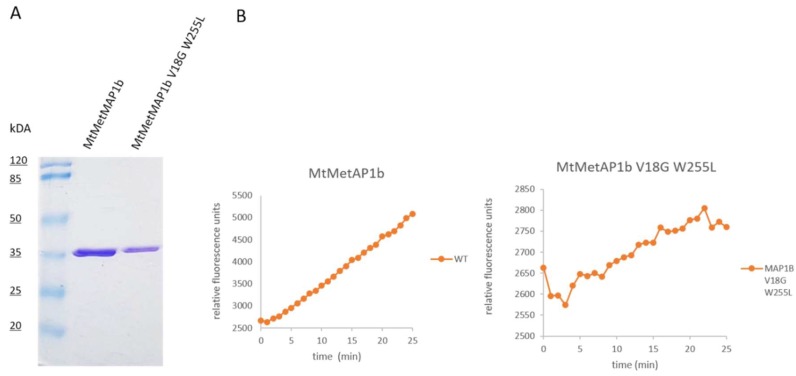
Enzymatic activity of recombinant *M. bovis* MetAP1c (MapB) and MetAP1c^V18G;W225L^ mutant. (**A**) Heterologous expressed and purified wt and mutant proteins in *E. coli* cells displayed on a 12 % SDS-PAGE. (**B**) The enzymatic activity of MetAP1c^WT^ and MetAP1c^V18G;W225L^ proteins was assayed with 0,2 µM protein in 50 mM Tris-HCL (pH 7.5) buffer containing 400 µM fluorogenic Met-MCA substrate and 1.5 mM MgCL2.

As we confirmed *mapB* is essential for *M. bovis* growth, we wanted to examine whether knockout mutants are rescued by expression of MAP with modified activity. It was previously reported that MapB^V18G^ and MapB^W255L^ were highly defective in their methionine aminopeptidase activity as the substitution W255L affects the catalytic pocket, whereas V18G affects the N-terminus extension of the protein [17]. We first designed these two mutation sequences separately and also a *mapB* sequence containing both point mutations: *mapB*^V18G^, *mapB*^W255L^ and *mapB*^V18G;W255L^. We expressed wild-type MapB and the MapB^V18G;W255L^ protein in *E. coli*, purified it and tested the activity *in vitro*. We found MapB^V18G;W255L^ had approximately 8% activity of the wt protein (Figure 5 and Supplementary Table S1). 

**Figure 6:**
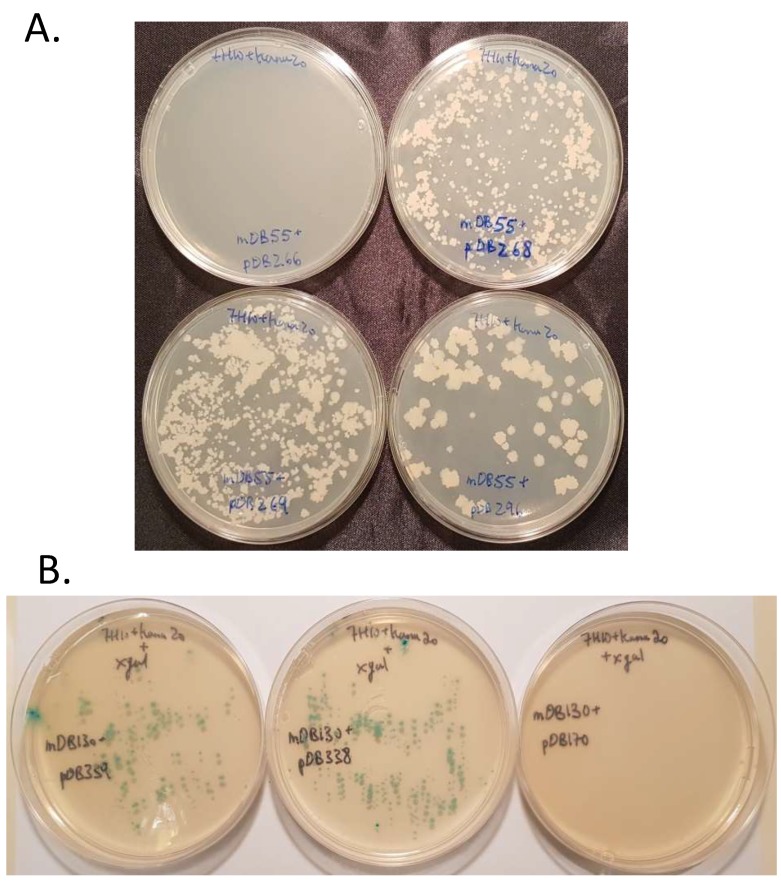
(**A**) when MapA is present, MapB can be replaced with MapB^V18G^ (pDB268, top right), MapB^W255L^ (pDB269, bottom left) or MapB^V18G;W255L^ (pDB296, bottom right), however not completely removed (pDB266, empty vector. Top left). (**B**) On the background of *mapA* deletion, MapB can still be replaced by MapB^V18G^ (pDB338, middle plate), MapB^W255L^ (pDB339, left plate) and not an empty vector (pDB170, right plate). All three plasmids (pDB170, 338, 339) also carry a *lacZ* gene for additional visual selection on top of kanamycin resistance.

We also cloned each gene separately (*mapB^V18G^, mapB^W255L^* and *mapB^V18G;W255L^*) into an *attp*-integrating vector with kanamycin resistance. The plasmids are designated pDB268 for *mapB*^V18G^; pDB269 for *mapB*^W255L^; pDB296 for *mapB*^V18G,W255L^. We attempted to replace the wt *mapB* gene at the *attb* site of mDB55 (where it is selected by streptomycin) by each one of the three mentioned kanamycin-selected vectors. As a control, we also used an empty *mapB*-null vector (expecting no colonies as the gene is vital) and a vector with a wild-type *mapB* copy (expecting multiple kanamycin resistant colonies) and indeed got the expected results. Interestingly, *mapB* could easily be replaced by any of the three mutated versions, including the *mapB^V18G;W255L^*, although the recombinant mutant protein had only traces of enzymatic activity. The colonies on all plates appeared after 3–4 weeks. This suggests that these rescued strains had no substantial growth defect in comparison to the growth of control strains (Figure 6, top). Randomly-selected colonies from all three *mapB* point mutations were examined by *mapB* sequencing and the unequivocal replacement of the wt gene by the mutated one was confirmed. 

To test if the bacteria could survive by means of the retained MapA activity, we performed a similar experiment with the double deletion mutant mDB130 (*ΔmapA:loxP; ΔmapB:zeocin; attp:mapB:hygromycin*). We attempted to replace the wt *mapB* gene at the *attP* site with the three low-activity *mapB* mutants, however for ease of identification of true transformants from background colonies, we used plasmids with LacZ activity (pDB338 for *mapB*^V18G^; pDB339 for *mapB*^W255L^; pDB340 for *mapB*^V18G,W255L^). Again, both *mapB^V18G^* and *mapB^W255L^* produced multiple viable colonies (Figure 6, bottom) which did not show any apparent growth defect in comparison to wt. 

However, only one blue colony was detectable with the extremely low-activity MetAPB variant *mapB^V18G;W255L^* and it was designated mDB180. The colony appeared after a 12 week incubation. Preliminary analysis suggested that the clone was a true gene-replacement mutant with a *mapB^V18G;W255L^* genotype as the colony was blue when plated on Xgal, the antibiotic resistance changed from hygromycin to kanamycin and a PCR pattern was consistent with a cassette exchange (Supplementary Figure S3). However, while PCR-based sequencing the *mapB* gene yielded equivocal results, whole genome sequencing found the mutant to be multiploid (up to 14 copies) in regards to the *lacZ*, *kanamycin^R^* and *mapB* genes. Among the *mapB* gene copies, approximately half of the genes correspond to the *mapB*^V18G,W255L^ genotype, while the other half of the sequences were *mapB*^W255L^, a genotype that was already shown to be fully viable. Thus, we conclude that on the background of *mapA* deletion, in contrast to wt background, a very-low activity MapB variant is insufficient to sustain bacterial growth. This suggested some, however not full, redundancy and mutual compensation between *mapA* and *mapB*. When *mapA* was present, the 8% activity of MapB^V18G,W255L^ was sufficient for viability and normal growth, whereas bacteria without *mapA* could retrieve no viable colonies. 

## 4. Discussion

As noted in the introduction, new drug targets are needed to combat tuberculosis and drug target validation is important in order to invest efforts into the development of effective drugs. Genes that code for essential enzymes are attractive targets since enzyme inhibition is a feasible drug-discovery task [18]. Therefore, correct identification of these genes/enzymes is important. 

The MetAP pathway is thought to be vital in all life forms [19,20,21] and this was specifically shown in many bacteria [5,6]. The basis for the essentiality of MetAP activity is not completely clear, however it appears that the removal of the Methionine is a prerequisite for other protein modifications such as N-myristoylation (probably only existing in eukaryotes) and N-alpha acetylation [22], and affects the stability and half-life of the protein [23,24]. As a first step for drug design, it might be important to gain more clarity about the essential gene, even in a small gene family. Here we provide unambiguous genetic proof that in fact *mapA* is dispensable, whereas *mapB* is essential. Our data are supported by a transposon library analysis previously performed [9] and contradicts a chemical-inhibition assay (Olaleyle et al.) [10]. We do not have an obvious explanation as to why the carefully performed, well-designed study by Olaleye suggested other essentiality results, however this may be another example where results from chemical inhibition may differ from those obtained in genetic deletion experiments [25]. It is not excluded that MapA is indeed more robust than MapB, however the protein substrates of MapB may be more important than those of MapA (if they differ at all, a matter which is unknown). Interestingly, most microorganisms have only one *map* gene which is usually a MetAP1a (meaning a homologue of Mtb *mapA* and not *mapB*) [26], suggesting that *mapA* could be sufficient for cell viability—a suggestion refuted in *M. bovis* by our findings.

It is unclear why it is that *mapB* is essential in Mtb, considering both MapB and MapA have MetAP activity (and that of MapA was shown to be even more robust than that of MapB) [8]. There are many hypothetical explanations for the differential requirement/essentiality of the two MetAP isoforms: Interactions with other proteins, a subset of proteins only affected by MapB and not by MapA, additional as-yet unidentified roles of these enzymes or a different affinity to the ribosome [27]. It was suggested that MetAPs played an important role in methionine turnover in the cell [28]. Still, it does not explain why one isoform is essential and the other not. Also, if this is the case, then methionine supplementation could perhaps salvage a full deletion mutant—however, a previous experiment reported the opposite [28]. 

Also, the relation between Peptide deformylase (PDF, encoded by *def, Rv0429c*) and MAP genes was not investigated. PDF, which removes the formyl group from the initiating methionine before the methionine itself is removed by MAP, is considered to be essential according to the analysis of a transposon library [9] and also by chemical inhibition in eukaryotic cells [23]. Since PDF activity is an absolute prerequisite for MetAP activity, the vital importance of MetAP explains the essentiality of PDF (*def*). Indeed, in previous experiments, a *def* deletion mutant in mycobacteria could only be created on the background of an inactivating mutation in formyl tRNA^fMet^ formyltransferase [29], making the removal of the formyl group unnecessary for the activity of MetAP.

## Supplementary Materials

The following are available online at https://www.mdpi.com/2073-4409/8/5/393/s1, Figure S1: Confirmation of a *mapA* deletion mutant. Figure S2: Failure to completely delete *mapB* from the genome. Figure S3: The exchange in *attb* cassettes from pDB271 (*mapB*^wt^, *hyg*^R^) to pDB340 (*mapB*^V18G,W255L^, *lacZ* ^+^ , *kanamycin*^R^) between mDB130 and mDB180. Supplementary Table S1: Plasmid table, primer table, mutant list.
