# Supplementary material for: MapB Protein is the Essential Methionine Aminopeptidase in Mycobacterium tuberculosis
Source: Cells. 2019 Apr 28;8(5):393. doi: 10.3390/cells8050393 (PMC6562599; doi:10.3390/cells8050393)
Supplement: Supplementary file 1 [file cells-08-00393-s001.pdf]

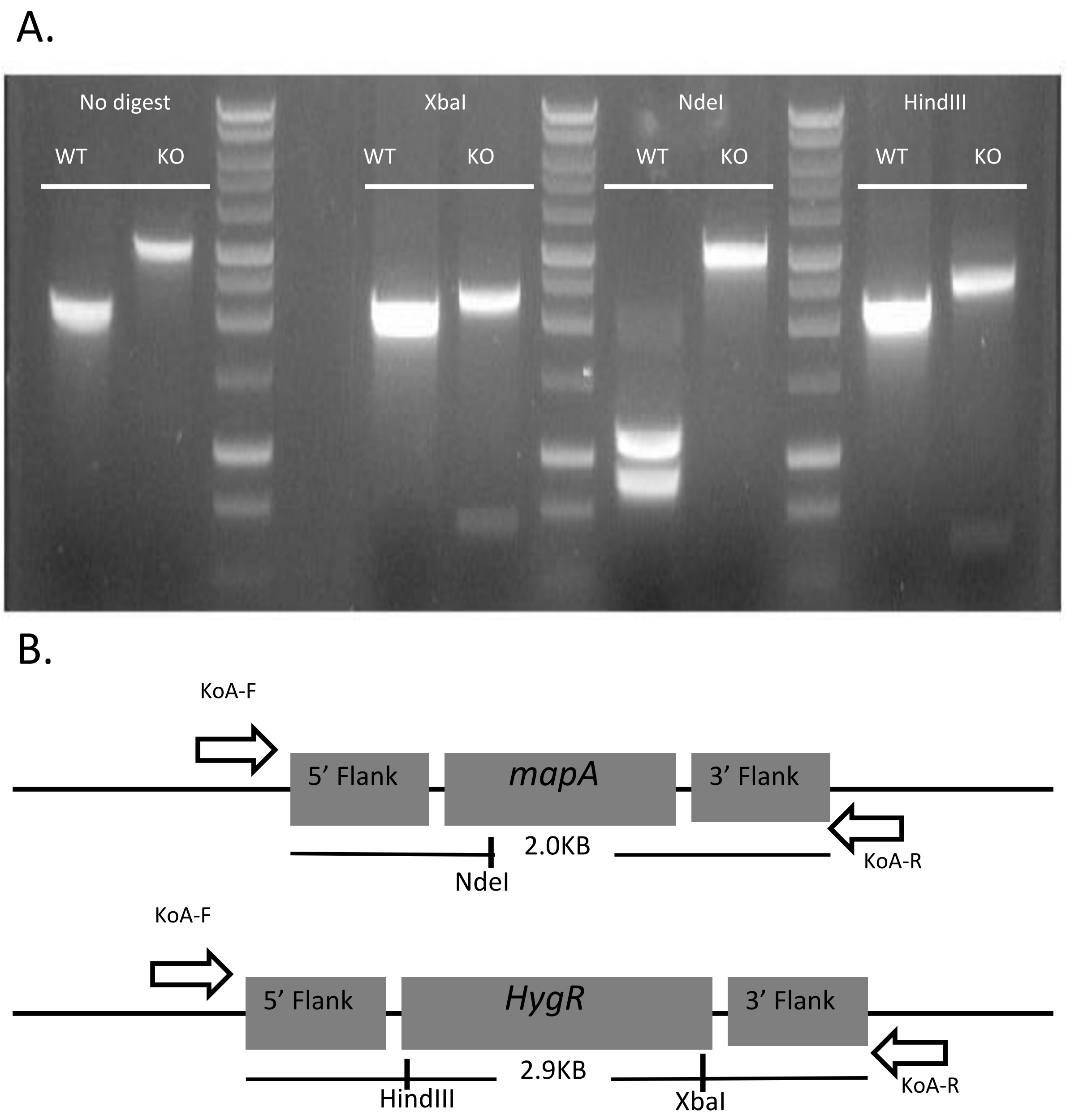

Supp. Fig 1: Confirmation of a *mapA* deletion mutant. one of the Hygromycin resistant colonies arising after infection with phDB20 was analyzed by using primers KoA-F, R. In wt the expected PCR product is of 2KB, whereas in a successful deletion mutant it is 2.9KB (Panel A, left). When the resulting product is digested by HindIII or XbaI, it is cut to 2.2KB and 0.7KB, whereas NdeI digestion does not affect it. In contrast, NdeI digestion cuts the wt product into 1.1 and 0.9KB fragments (Panel A, 3 rightmost couples). See Panel B for illustration of the genomic area in wt and in deletion mutant.

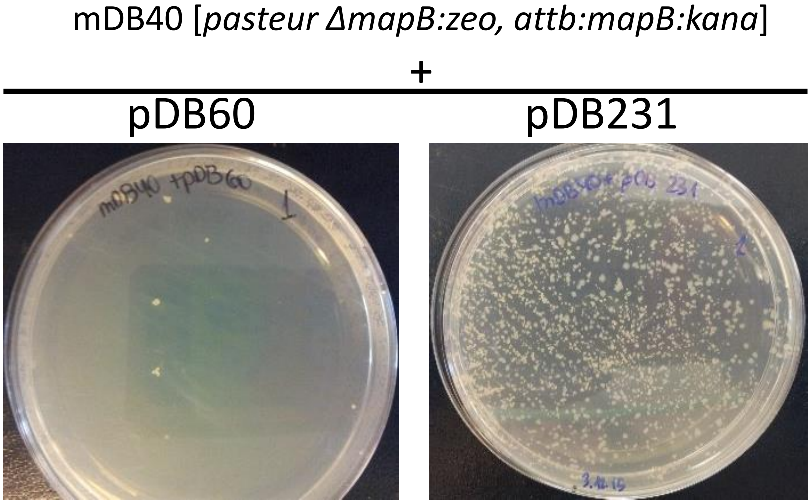

<u>Supp. Fig. 2</u>: <u>Failure to completely delete *mapB* from the genome</u>. mDB40 was electroporated by an empty, streptomycin-selected vector (pDB60) or a similar vector with another copy of *mapB* (pDB231). Only pDB231 yielded streptomycin-resistant colonies (Of the few colonies that grew on the pDB60 plates, all failed to grow in 7H9/streptomycin 20 μg/ml subculture).

A.
B.
C.

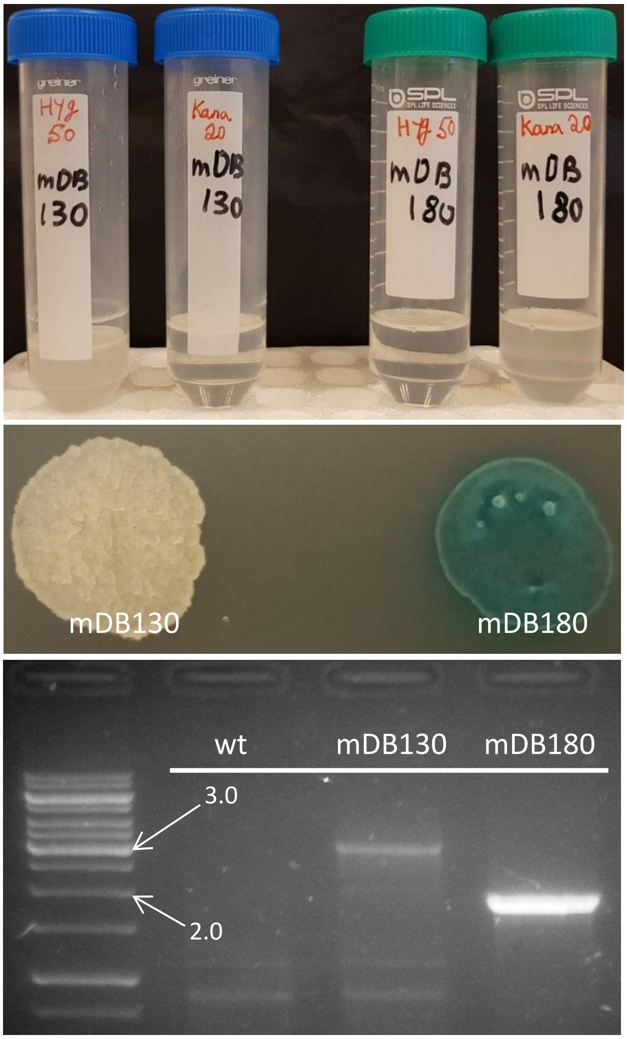

Supp. Fig 3: The exchange in *attb* cassettes from pDB271 (*mapB*^{wt}, *hyg*^{R}) to pDB340 (*mapB*^{V18G,W255L}, *lacZ*$^{+}$, *kanamycin*^{R}) between mDB130 and mDB180. **A**) mDB130 and mDB180 were grown in kanamycin 20 µg/ml or hygromycin 50 µg/ml. **B**) mDB130 and mDB180 were plated on X-gal containing 7H10 agar. **C**) PCR using primers 180-R1 and 180-F4. These primers produce no product on wt, 3.2 KB product on pDB271, and 1.9 KB product on pDB340.

Supplementary table 1: Plasmid table, primer table, mutant list:

Plasmid table:

Plasmid name	resistance	replication	genes
pDB116	kanamycin	attb integrating	*mapA*-AHT
pDB216--> phDB20	Hygromycin	OriE	Used to create phDB20, for deletion of *mapA*
pDB19	Zeocin	attb integrating	--
pDB249	Zeocin	attb integrating	*mapA*
pDB144	Kanamycin	attb integrating	*mapB*
pDB119-->phDB13	Zeocin	OriE	Used to create phDB13, for deletion of *mapB*
pDB60	Streptomycin	attb integrating	---
pDB218	Streptomycin	attb integrating	*mapA*
pDB231	Streptomycin	attb integrating	*mapB*
pDB261	Kanamycin	MF1 origin	*mapB*
pYUB412	Hygromycin	attb integrating	---
pDB271	Hygromycin	attb integrating	*mapB*
pDB266	Kanamycin	attb integrating	---
pDB267	Kanamycin	attb integrating	*mapB*
pDB268	Kanamycin	attb integrating	*mapB*^{V18G}
pDB269	Kanamycin	attb integrating	*mapB*^{W255L}
pDB296	Kanamycin	attb integrating	*mapB*^{V18G,W255L}
pDB338	Kanamycin	attb integrating	*mapB*^{V18G}, *lacZ*
pDB339	Kanamycin	attb integrating	*mapB*^{W255L}, *lacZ*
pDB340	Kanamycin	attb integrating	*mapB*^{V18G,W255L}. *lacZ*

Primer list:

Primer I	seq 5'- 3'	Primer II	seq 5'- 3'	product
koA-F	CTCATGCAGCGCAACTACGA	koA-R	CTCGTAGCGGCATCGTCATG	2000bp in wt; 2900bp in *ΔmapA*:hyg
mapA-F	CGAGTCGGTGATCCGCGAAT	mapA-R	CGTCCATTTGTCGTCGAGCA	547bp inside of *mapA*
KOc-E	ACGAAGTGCACGCAGTTGC	KOc-F	AAGTTGACCAGTGCCGTTC	350bp inside of Zeo^R
KOc-B	ACACGATGAACTCCAGCTCG	KOc-D-Rev	ACAATGCGACGACTGCACAA	2040bp in wt; 2100bp in *ΔmapB*:zeo
KOc-A	AGACACCTGAGGTCATCGAG	KOc-C	CTCGATGGTGAAGGTCATCC	590bp inside of *mapB*
Strp-R	TCACGCAACTGGTCCAGAAC	Strp-F	GGTGATCTCGCCTTTCACGTAG	1000bp inside of $strp^R$
attBsideR	GCTGGTGCAGTGAAGAGAAT	mapBmid-F	GTGACACCAACGCGACGTT	2067bp on pDB267,268, 269. 745bp on pDB231

Mutant list:

Name of mutant	origin	genotype
mDB25	BCG Pasteur+pDB116	*attb:mapA-AHT:kana*
mDB26	BCG Pasteur+pDB144	*attb:mapB:kana*

mDB40	mDB26+phDB13	*ΔmapB:zeo,attb:mapB:kan*
mDB55	mDB40+pDB231	*ΔmapB:zeo,attb:mapB:strep*
mDB59	mDB25+phDB20	*ΔmapA:hyg,attb:mapA-AHT:kana*
mDB77	mDB59+pDB19	*ΔmapA:hyg,attb:zeo*
mDB78	mDB59+pDB249	*ΔmapA:hyg,attb:mapA:zeo*
mDB103	mDB55+pDB261	*ΔmapB:zeo,attb:mapB:strep,MF1:mapB:kana*
mDB107	mDB103+pDB271	*ΔmapB:zeo,attb:mapB:hyg,MF1:mapB:kana*
mDB108	mDB103+pYUB412	*ΔmapB:zeo,attb:hyg,MF1:mapB:kana*
mDB110	mDB55+pDB268	*ΔmapB:zeo, attb: mapB*^{V18G}*;kana*
mDB111	mDB55+pDB269	*ΔmapB:zeo, attb: mapB*^{W255L}*;kana*
mDB146	mDB55+pDB296	*ΔmapB:zeo, attb: mapB*^{V18G,W255L}*;kana*
mDB174	mDB130+pDB339	*ΔmapA:lopX, ΔmapB:zeo, attb: mapB*^{W255L}*;kana:lacZ*
mDB175	mDB130+pDB338	*ΔmapA:lopX, ΔmapB:zeo, attb: mapB*^{V18G}*,kana:lacZ*
